# Albendazole-Induced SIRT3 Upregulation Protects Human Leukemia K562 Cells from the Cytotoxicity of MCL1 Suppression

**DOI:** 10.3390/ijms21113907

**Published:** 2020-05-30

**Authors:** Liang-Jun Wang, Li-Ren Liou, Yi-Jun Shi, Jing-Ting Chiou, Yuan-Chin Lee, Chia-Hui Huang, Po-Wei Huang, Long-Sen Chang

**Affiliations:** 1 Institute of Biomedical Sciences, National Sun Yat-Sen University, Kaohsiung 804, Taiwan; d052050011@student.nsysu.edu.tw (L.-J.W.); d052050012@student.nsysu.edu.tw (Y.-J.S.); d072050008@student.nsysu.edu.tw (J.-T.C.); d042050010@student.nsysu.edu.tw (Y.-C.L.); d042050004@student.nsysu.edu.tw (C.-H.H.); 2 Department of Surgery, Zuoying Branch of Kaohsiung Armed Forces General Hospital, Kaohsiung 813, Taiwan; allenliou@live.com (L.-R.L.); m870993@gmail.com (P.-W.H.); 3 Department of Biotechnology, Kaohsiung Medical University, Kaohsiung 807, Taiwan

**Keywords:** albendazole, leukemia, MCL1, SIRT3, apoptosis

## Abstract

Previous studies have shown that MCL1 stabilization confers cancer cells resistance to microtubule targeting agents (MTAs) and functionally extends the lifespan of MTA-triggered mitotically arrested cells. Albendazole (ABZ), a benzimidazole anthelmintic, shows microtubule-destabilizing activity and has been repositioned for cancer therapies. To clarify the role of MCL1 in ABZ-induced apoptosis, we investigated the cytotoxicity of ABZ on human leukemia K562 cells. Treatment with ABZ for 24 h did not appreciably induce apoptosis or mitochondrial depolarization in K562 cells, though it caused the mitotic arrest of K562 cells. ABZ-evoked p38 MAPK activation concurrently suppressed Sp1-mediated MCL1 expression and increased SIRT3 mRNA stability and protein expression. ABZ and A-1210477 (an MCL1 inhibitor) enhanced the cytotoxicity of ABT-263 (a BCL2/BCL2L1 inhibitor) to their effect on MCL1 suppression. Unlike ABZ, A-1210477 did not affect SIRT3 expression and reduced the survival of K562 cells. Overexpression of SIRT3 attenuated the A-1210477 cytotoxicity on K562 cells. ABZ treatment elicited marked apoptosis and ΔΨm loss in ABT-263-resistant K562 (K562/R) cells, but did not alter SIRT3 expression. Ectopic expression of SIRT3 alleviated the cytotoxicity of ABZ on K562/R cells. Collectively, our data demonstrate that ABZ-induced SIRT3 upregulation delays the apoptosis-inducing effect of MCL1 suppression on apoptosis induction in K562 cells.

## 1. Introduction

Microtubule dynamics and stability modulate cellular proliferation, trafficking, migration, and division [1,2]. Thus, microtubule-destabilizing (i.e., vincristine and nocodazole) and microtubule-stabilizing (i.e., paclitaxel and docetaxel) agents have been widely used for cancer therapy for the suppression of cell survival and growth [3]. The action of microtubule targeting agents (MTAs) causes G2/M cell cycle arrest, which activates the apoptotic death pathway in tumor cells [3,4]; however, the underlying mechanisms are poorly defined. Accumulating evidence has suggested that MTAs exert their cytotoxic effects by alteration in mitochondria function and cellular signaling, which are independent of the cell cycle phase [1,4,5].

The lethality of anthelmintic benzimidazole-based compounds on parasites is mediated through their microtubule-destabilizing activity [6,7]. Due to their low mammalian toxicity, benzimidazole anthelmintics, such as albendazole (ABZ), flubendazole (FUZ), and mebendazole (MBZ), have been repositioned for cancer therapies [8,9,10,11]. It has been suggested that the anti-cancer activity of benzimidazole anthelmintics is mediated through the disruption of microtubule organization. Nevertheless, several studies revealed that FUZ exerts its anti-cancer activity through the suppression of NFκB and STAT3 phosphorylation [12,13]. The induction of double-strand DNA breaks elucidates the MBZ-induced death of triple-negative breast cancer cells [14]. ABZ-induced TNF-α upregulation causes the apoptosis of leukemia U937 cells [15]. These findings highlight that some signaling pathways are coordinately involved in the apoptosis-inducing activity of benzimidazole anthelmintics. Previous studies have revealed that MCL1 degradation plays a central role in taxol or nocodazole-induced apoptosis in cancer cells, and thus the inhibition of MCL1 degradation confers a mechanism of resistance to taxol and nocodazole [16]. Haschka et al. [17] found that NOXA-mediated MCL1 degradation modulates the propensity of nocodazole-treated cancer cells to undergo mitotic apoptosis, whereas MCL1 stabilization extends the lifespan of mitotically arrested cells. Some studies have shown that taxol-treated chronic myeloid leukemia (CML) K562 cells show a delay in the induction of mitotic apoptosis [18]. Other studies have proposed that MCL1 is required for CML cell survival [19]. To clarify the function of MCL1 in maintaining the survival of K562 cells after treatment with benzimidazole anthelmintics, we have investigated the cytotoxicity of ABZ on K562 cells in this study. Our data showed that ABZ simultaneously induced SIRT3 upregulation and MCL1 downregulation in K562 cells, and that SIRT3 upregulation inhibited the cytotoxicity of ABZ-induced MCL1 suppression. Therefore, ABZ-induced MCL1 suppression did not immediately inhibit the survival of K562 cells after 24 h treatment. These findings suggested that SIRT3 played a role in extending the lifespan of ABZ-treated cells.

## 2. Results

**Figure 1:**
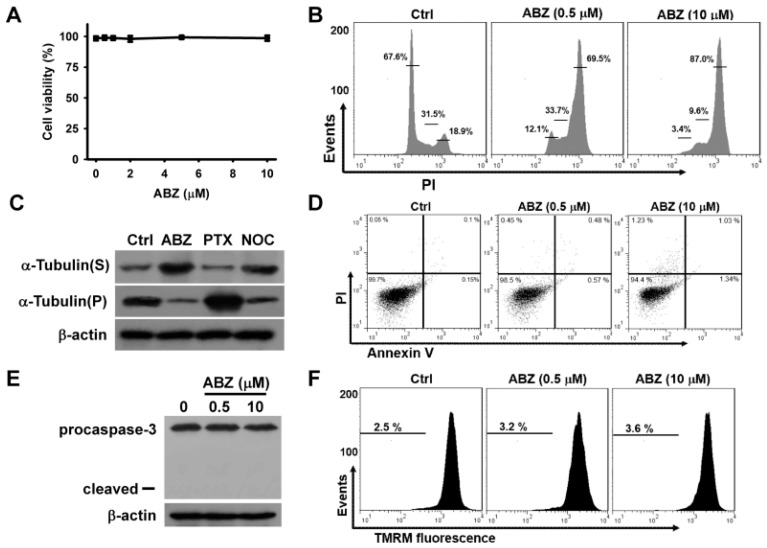
Cytotoxic effect of albendazole (ABZ) on K562 cells. Without specific indication, K562 cells were incubated with indicated ABZ concentrations for 24 h. (**A**) Concentration-dependent effect of ABZ on cell viability. K562 cells were incubated with indicated ABZ concentrations for 24 h. Cell viability was determined using MTT assay. Results are expressed as the percentage of cell viability relative to the control. Each value is the mean ± SD of three independent experiments with triplicate measurements. (**B**) Cell cycle analysis of ABZ-treated K562 cells. (**C**) Effect of ABZ, nocodazole, and paclitaxel on tubulin polymerization. K562 cells were treated with 0.25 μM nocodazole (NOC), 0.5 μM ABZ or 0.2 μM paclitaxel (PTX) for 24 h, respectively. Then, the cells were lysed and fractionated from cytosol (supernatant, S) to cytoskeletal (pellet, P) extracts. The extracts were subjected to Western blot analysis for α-tubulin and β-actin analysis. (**D**) Flow cytometry analyses of annexin V-PI double staining ABZ-treated cells. On the flow cytometric scatter graphs, the left lower quadrant represents remaining live cells. The right lower quadrant represents the population of early apoptotic cells. The right upper quadrant represents the accumulation of late apoptotic cells. (**E**) Western blot analyses of procaspase-3 degradation in ABZ-treated cells. (**F**) Effect of ABZ on ΔΨm in ABZ-treated cells.

Treatment with ABZ at quantities ranging from 0.5 μM to 10 μM for 24 h did not appreciably reduce the survival of K562 cells (Figure 1A) Treatment with either 0.5 or 10 μM ABZ notably increased the cell population arrested at G2/M phase of the cell cycle, while ABZ-treated cells did not show an appreciable increase in the cell population at sub-G1 area (Figure 1B). As shown in Figure 1C, both ABZ and nocodazole treatments suppressed tubulin polymerization, as evidenced by an increase in cytosolic α-tubulin (S fraction). The treatment of K562 cells with paclitaxel induced tubulin polymerization and thus increased the amount of α-tubulin in cytoskeletal (pellet, P) fractions. The ABZ-induced microtubule-destabilizing effect caused a G2/M cell cycle arrest. An annexin V/PI staining assay showed that treatment with ABZ did not appreciably induce apoptosis in K562 cells (Figure 1D). Additionally, ABZ-treated cells did not show the degradation of procaspase-3 or the loss of mitochondrial membrane potential (ΔΨm) (Figure 1E,F). These results indicate that mitotic arrest induced by ABZ did not immediately activate the apoptotic pathway in K562 cells.

**Figure 2:**
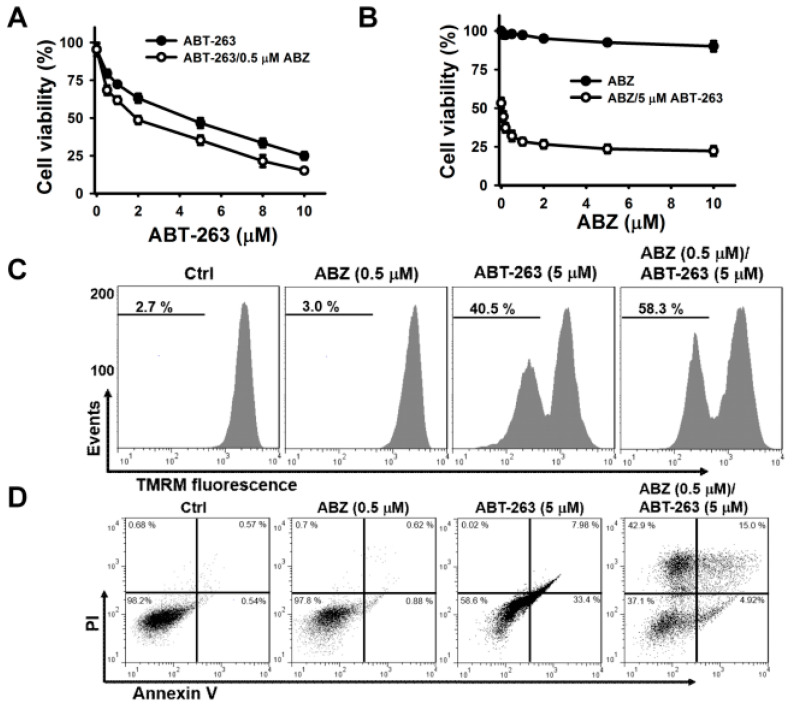

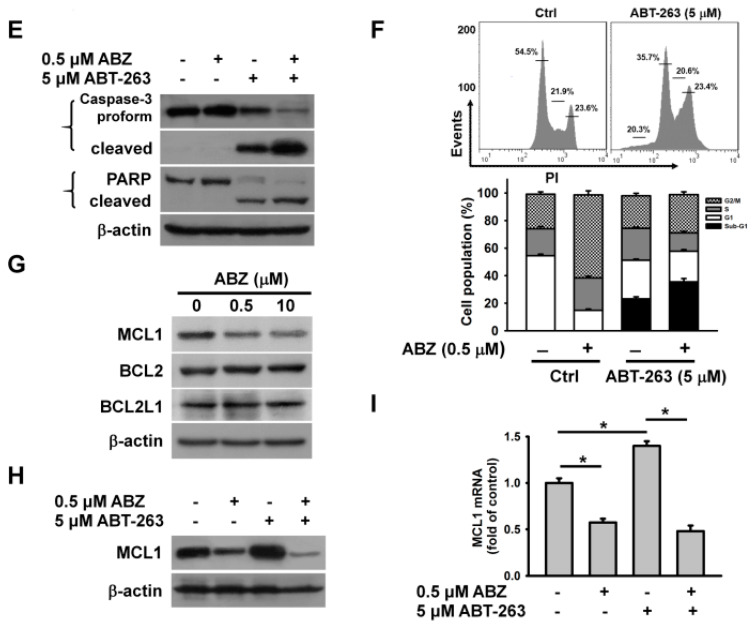
ABT-263 induced apoptosis of ABZ-treated K562 cells. Without specific indication, K562 cells were incubated with 0.5 μM ABZ, 5 μM ABT-263, or the combination of 0.5 μM ABZ and 5 μM ABT-263 for 24 h. (**A**) ABZ enhanced the cytotoxicity of ABT-263. K562 cells were incubated with 0.5 μM ABZ and indicated ABT-263 concentrations for 24 h. (**B**) ABT-263 enhanced the cytotoxicity of ABZ. K562 cells were incubated with 5 μM ABT-263 and indicated ABZ concentrations for 24 h. (**C**) Analysis of ΔΨm in ABZ-, ABT-263-, and ABZ/ABT-263-treated K562 cells. Moreover, ΔΨm was analyzed by flow cytometry. (**D**) Annexin V-PI double staining of ABZ-, ABT-263-, and ABZ/ABT-263-treated K562 cells. (**E**) The cleavage of procaspase-3 and PARP in ABT-263- and ABZ/ABT-263-treated K562 cells. (**F**) Cell cycle analysis of ABZ-, ABT-263-, and ABZ/ABT-263-treated K562 cells. (Top panel) Cell cycle distribution of ABT-263-treated cells. (Bottom panel) Quantitative analyses of cell cycle distribution in K562 cells treated with ABZ, ABT-263, and ABZ/ABT-263. (**G**) Effect of ABZ on MCL1, BCL2, and BCL2L1 expression in K562 cells. K562 cells were treated with indicated ABZ concentrations for 24 h. (**H**) ABZ suppressed ABT-263-induced MCL1 upregulation. (**I**) ABZ suppressed ABT-263-induced increased MCL1 mRNA level. The level of MCL1 mRNA was analyzed by qPCR (mean ± SD, * p < 0.05).

Previous studies have shown that benzimidazole anthelmintics potentiate the cytotoxicity of BH3 mimetic ABT-263 (a BCL2/BCL2L1 inhibitor) on non-small cell lung cancer [20]. Haschka et al. [17] found that BH3 mimetic ABT-737 (a BCL2/BCL2L1 inhibitor) increasingly triggers the apoptosis of mitotic cells, arrested by nocodazole induction. BH3 mimetics have been reported to induce cell death through the activation of the intrinsic apoptosis pathway [17,20]. To examine whether the inability of ABZ to induce the death of K562 cells was associated with defects in activating intrinsic apoptosis machinery, the cytotoxic effect of ABT-263 on ABZ-treated and -untreated K562 cells were analyzed. Since K562 cells exposed to 0.5 μM ABZ showed a marked G2/M cell cycle arrest, we used this concentration of ABZ to investigate the effect of combination with ABT-263 on apoptosis induction over 24-h. Treatment with ABT-263 plus ABZ induced the death of K562 cells at a higher rate compared with ABT-263 alone (Figure 2A). ABT-263 also promoted the death of ABZ-treated cells (Figure 2B). In comparison with a singular treatment of ABT-263 or ABZ, combinatorial treatment of ABT-263 and ABZ increased the loss of ΔΨm (Figure 2C) and apoptosis (Figure 2D) in K562 cells. Consistently, in comparison with either agent alone, the combination of ABT-263 and ABZ markedly increased the production of cleaved caspase-3 and PARP in K562 cells (Figure 2E). Co-treatment with ABT-263 caused an increase in the cell population at sub-G1 area, accompanied by a decrease in the cell population at G2/M phase in ABZ-treated cells (Figure 2F). This revealed that ABT-263 preferably elicited the apoptosis of mitotically arrested cells. Similarly, co-treatment with ABT-737 forces nocodazole-treated cancer cells to undergo cell death during M-arrest [17]. Since previous research has revealed that MCL1 degradation crucially determines the susceptibility of MTA-treated cells to undergoing apoptosis [16,17], MCL1 expression in ABZ-treated K562 cells was thus examined. ABZ treatment caused MCL1 downregulation, but the levels of BCL2 and BCL2L1 proteins remained unchanged (Figure 2G). Moreover, combined treatment with ABZ mitigated the ABT-263-elicited upregulation of MCL1 protein and mRNA expression (Figure 2H,I). It has been suggested that MCL1 expression confers tumor cells resistance to ABT-263 [21,22]. These findings indicate that ABZ-induced MCL1 downregulation increases the cytotoxicity of ABT-263, and suggest that some survival pathways counteract the ability of ABZ-induced MCL1 suppression to induce the apoptosis of K562 cells.

**Figure 3:**
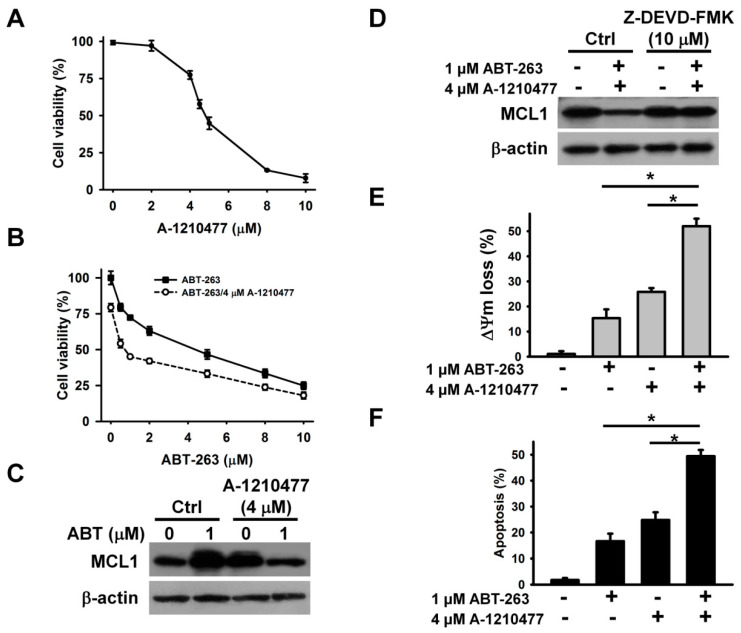
A-1210477 enhanced the cytotoxicity of ABT-263. (**A**) The cytotoxicity of A-1210477 on K562 cells. K562 cells were treated with indicated A-1210477 concentrations for 24 h. (**B**) Effect of A-1210477 on the cytotoxicity of ABT-263 on K562 cells. K562 cells were treated with 4 μM A-1210477 and indicated ABT-263 concentrations for 24 h. (**C**) Western blot analyses of MCL1 expression in A-1210477-, ABT-263-, and A-1210477/ABT-263-treated cells. K562 cells were treated with 1 μM ABT-263 and/or 4 μM A-1210477 for 24 h. (**D**) Effect of caspase-3 inhibitor on MCL1 expression in A-1210477/ABT-263-treated cells. K562 cells were pretreated with 10 μM Z-DEVD-FMK for 1 h, and then incubated with ABT-263 plus A-1210477 for 24 h. (**E**) Effect of A-1210477, ABT-263, or A-1210477/ABT-263 on ΔΨm in K562 cells. (**F**) Effect of A-1210477, ABT-263, or A-1210477/ABT-263 on apoptosis induction in K562 cells. Apoptosis was assessed in triplicate by annexin V-PI double staining followed by flow cytometry, and percentage apoptosis is shown as percentage of annexin V-positive cells. Data represent mean ± SD (* p < 0.05).

To further explore whether MCL1 suppression alone can cause the death of K562 cells, we examined the cytotoxicity of A-1210477 (an MCL1 inhibitor) on K562 cells. A-1210477 dose-dependently decreased the survival of K562 cells after 24 h of treatment (Figure 3A). Treatment with 4 μM A-1210477 caused an approximately 25% loss in K562 cell viability. To examine the enhancement of ABT-263 cytotoxicity when combined with A-1210477, the sub-lethal concentration of A-1210477 was used. Co-treatment with 4 μM A-1210477 markedly increased the cytotoxicity of ≤ 1 μM ABT-263 on K562 cells (Figure 3B). This finding aligns with previous studies, which show that A-1210477 synergizes with ABT-199 (a BCL2 inhibitor), to kill a variety of cancer cell lines [23]. Either A-1210477 or ABT-263 treatment increased MCL1 protein expression in K562 cells (Figure 3C). Similarly, previous studies have shown that ABT-263 upregulates MCL1 expression in cancer cells [21], while A-1210477 increases MCL1 accumulation, due to the inhibition of NOXA-mediated MCL1 degradation [23]. Nevertheless, co-treatment with A-1210477 and ABT-263 reduces MCL1 expression in K562 cells. Studies by Ryu et al. [24] have reported a caspase-mediated MCL1 cleavage in ABT-737-treated leukemia cells. Consistent with these findings, the present study found that treatment with a caspase-3 inhibitor restored MCL1 expression (Figure 3D). Compared to either A-1210477 or ABT-263, the combinatorial treatment increased the loss of ΔΨm and apoptosis in K562 cells (Figure 3E,F).

**Figure 4:**
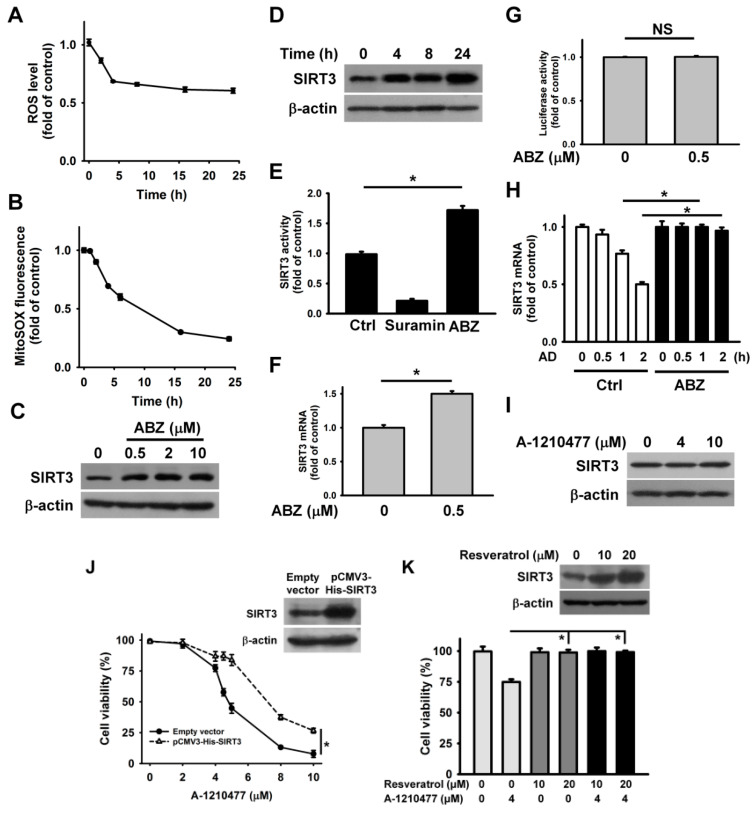
ABZ induced SIRT3 upregulation in K562 cells. Without specific indication, K562 cells were treated with 0.5 μM ABZ for 24 h. (**A**) ABZ induced a reduction in ROS generation. K562 cells were incubated with ABZ for indicated time periods. Results were shown as fold-increase in fluorescence intensity compared with the control group. Each value is the mean ± SD of three independent experiments with triplicate measurements. ROS was quantified by the fluorescence plate reader. (**B**) Measurement of mitochondrial ROS generation using mitochondrial superoxide probe MitoSOX Red. K562 cells were incubated with ABZ for indicated time periods. The data represent the mean ± SD. Effect of ABZ induced SIRT3 expression in (**C**) concentration- and (**D**) time-dependent manners. (**E**) Effect of ABZ on SIRT3 deacetylase activity in K562 cells. A control experiment was also conducted using 5 mM suramin (a SIRT inhibitor), provided in the kit. Data represent mean ± SD (* p < 0.05). (**F**) Analysis of SIRT3 mRNA levels in ABZ-treated cells. The values represent averages of three independent experiments with triplicate measurements (mean ± SD, * p < 0.05). (**G**) Effect of ABZ on the luciferase activity of SIRT3 promoter construct. SIRT3 promoter construct-transfected cells were treated with ABZ for 24 h and then harvested for measuring luciferase activity (NS, statistically insignificant). (**H**) Effect of ABZ on SIRT3 mRNA stability. Cells were treated with or without ABZ for 24 h, and then incubated with 10 μg/mL actinomycin D (AD) for the indicated time periods. The level of SIRT3 mRNA was analyzed by qPCR. ABZ-untreated and ABZ-treated cells without actinomycin D treatment were used as control (mean ± SD, * p < 0.05). (**I**) Effect of A-1210477 on SIRT3 expression in K562 cells. K562 cells were treated with indicated A-1210477 concentrations for 24 h. (**J**) SIRT3 overexpression increased the viability of A-1210477-treated cells. After transfection with an empty vector or pCMV3-His-SIRT3 for 24 h, the transfected cells were treated with indicated A-1210477 concentrations for 24 h. Cell viability was determined using MTT assay (mean ± SD, * p < 0.05). (Inset) Western blot analysis of SIRT3 expression in pCMV3-His-SIRT3-transfected cells. (**K**) Effect of resveratrol on the viability of A-1210477-treated cells. K562 cells were treated with indicated resveratrol concentrations for 1 h, and then incubated with 4 μM A-1210477 for 24 h (mean ± SD, * p < 0.05). (Inset) Effect of resveratrol on SIRT3 expression.

The above results indicate that MCL1 inhibition by A-1210477 and MCL1 downregulation by ABZ enhance ABT-263 cytotoxicity. Unlike A-1210477, ABZ-induced MCL1 suppression does not induce the death of K562 cells. These observations likely suggest that ABZ evokes a pro-survival pathway in K562 cells. Recent studies have shown that ABZ-induced SIRT3 suppression causes the generation of mitochondrial ROS, which subsequently elicits apoptosis in leukemia cells [15]. Astonishingly, a sustained decrease in intracellular ROS and mitochondrial ROS levels was observed in K562 cells after ABZ treatment (Figure 4A,B). Taking into account that SIRT3 modulates the activity of SOD2 on scavenging mitochondrial ROS [25], we analyzed SIRT3 expression in ABZ-treated cells. ABZ treatment caused a concentration and time dependent increase in SIRT3 protein expression (Figure 4C,D). Consistently, the measurement of SIRT3 deacetylase activity showed that ABZ treatment increased the SIRT3 activity (Figure 4E). An increase in the SIRT3 mRNA level was noted in ABZ-treated K562 cells (Figure 4F), but ABZ treatment did not affect SIRT3 promoter luciferase activity (Figure 4G). We thus hypothesized that ABZ treatment stabilized SIRT3 mRNA. To test this hypothesis, SIRT3 mRNA half-life in untreated control cells and ABZ-treated cells were analyzed. Transcription blocking with actinomycin D showed that ABZ treatment caused a reduction in SIRT3 mRNA decay (Figure 4H), revealing that ABZ post-transcriptionally upregulates SIRT3 expression. Notably, A-1210477 treatment did not affect SIRT3 expression (Figure 4I). The overexpression of SIRT3 attenuated A-1210477-induced cell death (Figure 4J), suggesting that ABZ-induced SIRT3 upregulation counteracts the apoptosis-inducing effect of MCL1 suppression in K562 cells. Previous studies have shown that resveratrol is a SIRT3 activator [26]. Thus, we analyzed the effect of resveratrol on A-1210477 cytotoxicity. As shown in Figure 4K, resveratrol restored the viability of A-1210477-treated K562 cells. Furthermore, resveratrol also induced SIRT3 upregulation. These results indicate that SIRT3 activation attenuates the cytotoxicity of MCL1 suppression.

**Figure 5:**
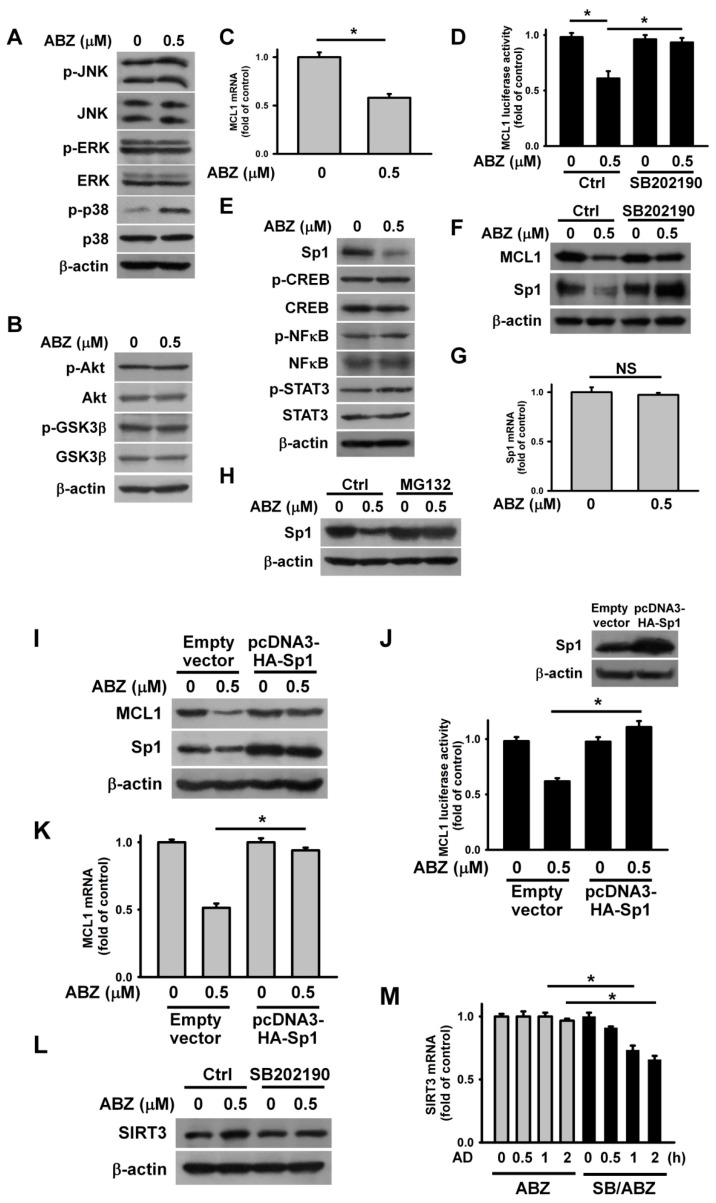
ABZ-induced p38 MAPK activation suppressed Sp1-mediated MCL1 transcription and increased SIRT3 mRNA stability. Without specific indication, K562 cells were incubated with 0.5 μM ABZ for 24 h. (**A**) Effect of ABZ on MAPK phosphorylation. (**B**) Effect of ABZ on Akt and GSK3β phosphorylation. (**C**) Analysis of MCL1 mRNA levels in ABZ-treated cells. The values represent averages of three independent experiments with triplicate measurements (mean ± SD, * p < 0.05). (**D**) Effect of ABZ and SB202190 on the luciferase activity of MCL1 promoter construct in K562 cells and ABZ-treated K562 cells. MCL1 promoter construct-transfected cells were pretreated with or without 10 μM SB202190 for 1 h, and then incubated with 0.5 μM ABZ for 24 h (mean ± SD, * p < 0.05). (**E**) Effect of ABZ on Sp1 expression and the levels of p-CREB/p-STAT3/p-NFκB. (**F**) Effect of SB202190 on Sp1 and MCL1 expression in ABZ-treated cells. K562 cells were pretreated with 10 μM SB202190 for 1 h, and then incubated with 0.5 μM ABZ for 24 h. (**G**) Effect of ABZ on Sp1 mRNA levels in ABZ-treated cells. The level of Sp1 mRNA was analyzed by qPCR (NS, statistically insignificant). (**H**) Effect of MG132 on Sp1 expression in ABZ-treated cells. K562 cells were pretreated with 1 μM MG132 for 1 h, and then incubated with 0.5 μM ABZ for 24 h. Effect of Sp1 overexpression on MCL1 protein expression (**I**), MCL1 promoter luciferase activity (**J**) and MCL1 mRNA levels (**K**) in ABZ-treated cells. After transfection with an empty vector or pcDNA3-HA-Sp1 for 24 h, the transfected cells were treated with 0.5 μM ABZ for 24 h. Overexpression of Sp1 abolished the suppressive effect of ABZ on MCL1 promoter luciferase activity (mean ± SD, * p < 0.05). The level of MCL1 mRNA was analyzed by qPCR (mean ± SD, * p < 0.05). (**L**) Effect of SB202190 on SIRT3 expression in ABZ-treated cells. K562 cells were pretreated with 10 μM SB202190 for 1 h, and then incubated with 0.5 μM ABZ for 24 h. (**M**) Effect of SB202190 on the stability of SIRT3 mRNA in ABZ-treated cells. ABZ-untreated and ABZ-treated cells without actinomycin D treatment were used as control (mean ± SD, * p < 0.05).

Some studies have revealed that Akt and MAPK actively regulate MCL1 transcription, and Akt-modulated GSK3β phosphorylation is crucial for MCL1 degradation [27,28]. Thus, we sought to analyze the phosphorylation of MAPK, Akt, and GSK3β in ABZ-treated cells. ABZ treatment increased p38 MAPK phosphorylation, but did not alter p-ERK or p-JNK levels (Figure 5A). Meanwhile, neither the phosphorylation of Akt nor GSK3β was affected by ABZ treatment (Figure 5B). The treatment of ABZ reduced MCL1 mRNA levels and promoter luciferase activity (Figure 5C,D). These results suggest that ABZ downregulates MCL1 expression via inhibition of MCL1 transcription. Previous studies have reported the involvement of the transcriptional factors Sp1, STAT3, NFκB, and CREB in MCL1 transcription [29,30,31]. ABZ treatment induced Sp1 downregulation, but did not affect CREB, STAT3, or NFκB phosphorylation or expression (Figure 5E). The inhibition of p38 MAPK by SB202190 increased MCL1 and Sp1 expression in ABZ-treated cells (Figure 5F). Furthermore, SB202190 mitigated the inhibitory effect of ABZ on MCL1 promoter luciferase activity (Figure 5D). ABZ treatment did not significantly affect the levels of Sp1 mRNA (Figure 5G). The inhibition of proteasome by MG132 restored Sp1 expression (Figure 5H), suggesting that ABZ induces Sp1 degradation. The ectopic expression of Sp1 eliminated the effect of ABZ on the suppression of MCL1 expression and promoter luciferase activity (Figure 5I–K). These results emphasize that ABZ inhibits MCL1 transcription through p38 MAPK-mediated Sp1 degradation. In agreement, the nucleotide sequence of MCL1 promoter region contains a Sp1-binding site at positions from −167 to −159 [29]. Notably, pretreatment with SB202190 decreased SIRT3 expression and mRNA stability in cells exposed to ABZ (Figure 5L,M). These findings suggested that ABZ-elicited p38 MAPK activation causes MCL1 suppression and SIRT3 upregulation concurrently.

**Figure 6:**
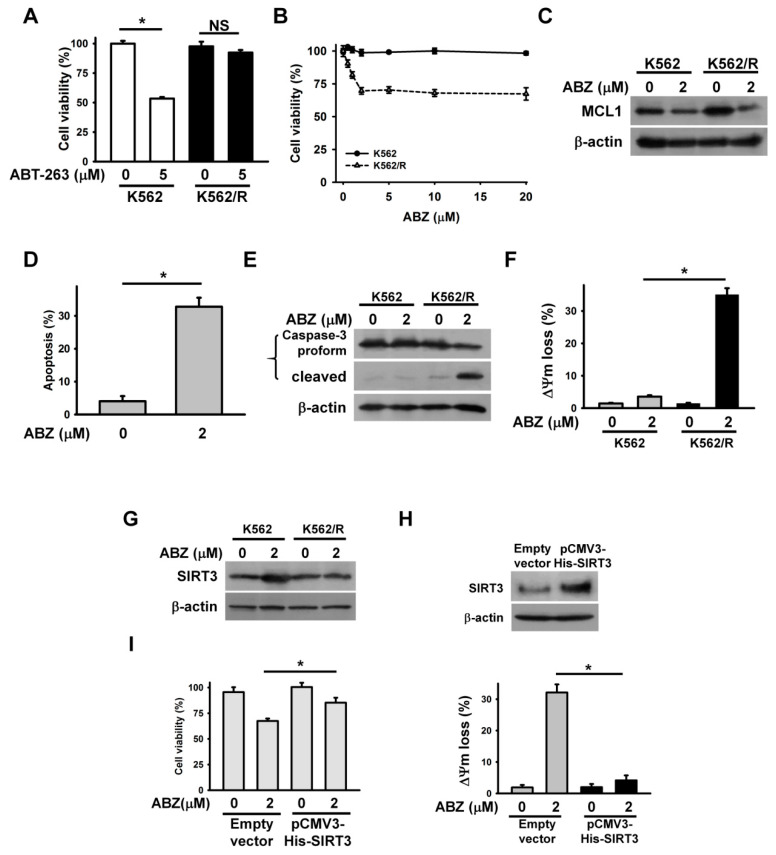
The cytotoxic effect of ABZ on K562/R cells. Without specific indication, K562 and K562/R cells were treated with 2 μM ABZ for 24 h. (**A**) Effect of ABT-263 on the viability of K562 and K562/R cells. K562 and K562/R cells were treated with 5 μM ABT-263 for 24 h. Cell viability was determined using MTT assay (* p < 0.05; NS, statistically insignificant). (**B**) Effect of ABZ on the viability of K562 and K562/ABT-R cells. K562 and K562/ABT-R cells were treated with indicated ABZ concentrations for 24 h. (**C**) Effect of ABZ on MCL1 expression in K562/R cells. (**D**) ABZ induced apoptosis of K562/R cells. Apoptosis was assessed in triplicate by annexin V-PI double staining, followed by flow cytometry, and percentage apoptosis is shown as percentage of annexin V-positive cells. Data represent mean ± SD (* p < 0.05). (**E**) ABZ induced the cleavage of procaspase-3 in K562/R cells. (**F**) ABZ induced the loss of ΔΨm in K562/R cells. (**G**) Effect of ABZ on SIRT3 expression in K562/R cells. (**H**) SIRT3 overexpression inhibited ABZ-induced ΔΨm loss in K562/R cells. (Top panel) SIRT3 expression in pCMV3-His-SIRT3-transfected K562/R cells. (Bottom panel) Effect of ABZ on ΔΨm of empty vector- and pCMV3-His-SIRT3-transfected K562/R cells (mean ± SD, * p < 0.05). (**I**) SIRT3 overexpression increased the viability of ABZ-treated K562/R cells (mean ± SD, * p < 0.05).

Accumulated evidence shows that MCL1 expression confers tumor cells resistance to ABT-263 [21,22]. For this reason, the cytotoxicity of ABZ on ABT-263-resistant cancer cells is an interesting and relevant topic in the field of cancer research. ABT-263-resistant K562 cells (K562/R) were prepared by continuous exposure of parental K562 cells to 5 μM ABT-263. The K562/R cells remained alive after ABT-263 treatment for 24 h (Figure 6A). Notably, ABZ showed higher cytotoxicity on K562/R cells than on parental cells (Figure 6B). The treatment of K562/R cells with 2 μM ABZ for 24 h caused an approximate 25% loss in cell viability. Thus, we used this concentration of ABZ to investigate its cytotoxicity on K562/R cells. Immunoblotting showed that K562/R cells had higher MCL1 expression than K562 cells (Figure 6C). Treatment with ABZ similarly downregulated MCL1 in parental and K562/R cells. An annexin V-PI staining assay showed that ABZ induced the apoptosis of K562/R cells (Figure 6D). Similarly, ABZ-treated K562/R cells produced cleaved caspase-3 (Figure 6E) and the dissipation of ΔΨm (Figure 6F). In contrast to K562 cells, K562/R cells did not show SIRT3 upregulation after ABZ treatment (Figure 6G). The ectopic expression of SIRT3 reduces the loss of ΔΨm in ABZ-treated K562/R cells (Figure 6H) and increased the survival of ABZ-treated K562/R cells (Figure 6I). These results indicate that MCL1 suppression elicits the apoptosis of ABZ-treated K562/R cells when SIRT3 expression is not altered.

**Figure 7:**
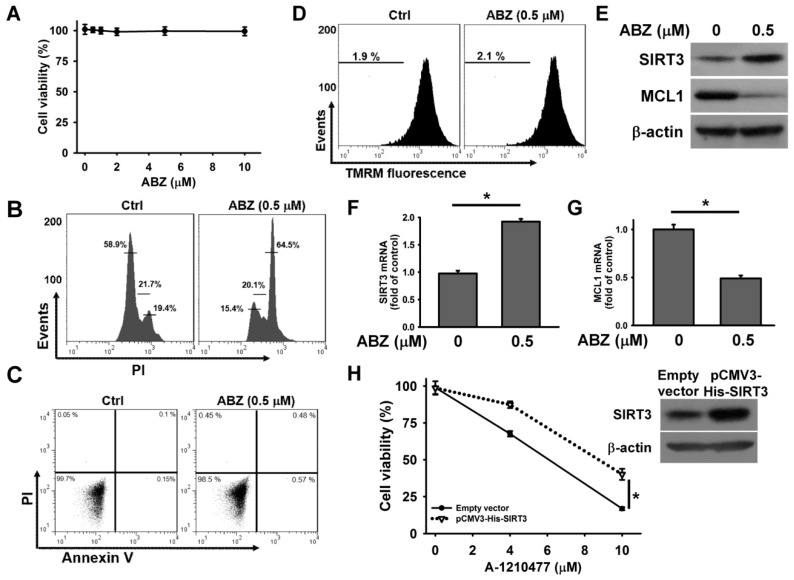
SIRT3 upregulation protected MEG-01 cells from the cytotoxicity of ABZ-induced MCL1 downregulation. Without specific indication, MEG-01 cells were treated with 0.5 μM ABZ for 24 h. (**A**) Concentration-dependent effect of ABZ on cell viability. MEG-01 cells were incubated with indicated ABZ concentrations for 24 h. Cell viability was determined using MTT assay. (**B**) Cell cycle analysis of ABZ-treated MEG-01 cells. (**C**) Flow cytometry analyses of annexin V-PI double staining ABZ-treated cells. (**D**) Effect of ABZ on ΔΨm in ABZ-treated cells. (**E**) Western blot analyses of SIRT3 and MCL1 expression in ABZ-treated cells. Analysis of (**F**) SIRT3 mRNA and (**G**) MCL1 mRNA levels in ABZ-treated cells (mean ± SD, * p < 0.05). (**H**) SIRT3 overexpression increased the viability of A-1210477-treated MEG-01 cells. After transfection with an empty vector or pCMV3-His-SIRT3 for 24 h, the transfected cells were treated with indicated A-1210477 concentrations for 24 h. Cell viability was determined using MTT assay (mean ± SD, * p < 0.05). (Inset) Western blot analysis of SIRT3 expression in pCMV3-His-SIRT3-transfected cells.

To examine whether SIRT3 also inhibited the cytotoxic effect of ABZ-induced MCL1 suppression in other cell lines, we analyzed the cytotoxicity of ABZ on human leukemia MEG-01 cells. As shown in Figure 7A, ABZ did not appreciably reduce the survival of MEG-01 after 24 h treatment. Treatment with 0.5 μM ABZ notably increased the cell population arrested at G2/M phase of the cell cycle, but did not induce apoptosis and ΔΨm loss in MEG-01 cells (Figure 7B–D). ABZ treatment increased SIRT3 expression and decreased MCL1 expression in MEG-01 cells (Figure 7E). Consistently, ABZ-treated MEG-01 cells showed an increase in SIRT3 mRNA expression and a reduction in MCL1 mRNA expression (Figure 7F,G). Overexpression of SIRT3 attenuated the A-1210477-induced death of MEG-01 cells (Figure 7H). These findings suggest that SIRT3 upregulation protects MEG-01 cells from the cytotoxicity of MCL1 suppression.

## 3. Discussion

**Figure 8:**
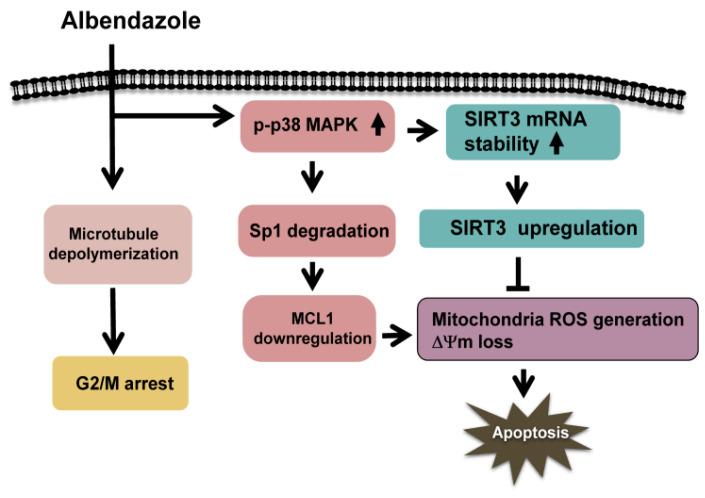
Signaling pathways elucidate the protective effect of SIRT3 upregulation on the cytotoxicity of ABZ-induced MCL1 suppression in K562 cells. ABZ treatment simultaneously induces microtubule depolymerization, MCL1 downregulation, and SIRT3 upregulation in K562 cells. ABZ inhibits MCL1 transcription through p38 MAPK-mediated Sp1 degradation. Meanwhile, ABZ-induced p38 MAPK activation increases the stability of SIRT3 mRNA and SIRT3 protein expression in K562 cells. SIRT3 upregulation delays ABZ-induced apoptosis in K562 cells when ABZ inhibits MCL1 expression. Thus, SIRT3 expression modulates the capability of ABZ to induce apoptosis in K562 cells.

Our data show that ABZ inhibits MCL1 transcription in K562 cells through p38 MAPK-mediated Sp1 degradation, but MCL1 downregulation does not appreciably inhibit the survival of ABZ-treated K562 cells (Figure 8). Meanwhile, ABZ-induced p38 MAPK activation increases the stability of SIRT3 mRNA and SIRT3 protein expression in K562 cells (Figure 8). ABZ induces MCL1 downregulation and apoptosis in K562/R cells, whereas ABZ is unable to upregulate SIRT3 expression in K562/R cells. The overexpression of SIRT3 alleviates the cytotoxicity of A-1210477 (an MCL1 inhibitor) on K562 cells and the cytotoxicity of ABZ on K562/R cells. Collectively, these results indicate that SIRT3 upregulation counteracts the apoptosis-inducing effect of MCL1 suppression in ABZ-treated K562 cells (Figure 8). Similarly, the protective effect of SIRT3 on reducing the cytotoxicity of MCL1 suppression is noted in MEG-01 cells. Studies by Sundaresan et al. [32] revealed that SIRT3 partly impedes the mitochondrial translocation of BAX and thus increases the survival of cardiomyocytes in stress situations, which may elucidate the protective effect of SIRT3 on ABZ-treated K562 cells. On the other hand, Mallick et al. [33] reported that A-1210477 exerts off-target effects that induce the apoptosis of cancer cells, in addition to targeting MCL1. This finding might describe the inability of SIRT3 overexpression to fully eliminate A-1210477 cytotoxicity on K562 cells (Figure 4J). Co-treatment with ABT-263 (a BCL2/BCL2L1 inhibitor) causes ΔΨm loss and apoptosis in ABZ-treated K562 cells, which indicates that ABZ-induced SIRT3 upregulation could not repress the cytotoxicity triggered by the inhibition of BCL2/BCL2L1. Suppression of the anti-apoptotic proteins MCL1, BCL2, and BCL2L1 by BH3 mimetics has been well-known to induce the BAX/BAK-modulated mitochondrial death pathway of pro-apoptotic proteins in cancer cells [34]. Considering that SIRT3 plays a role in maintaining normal mitochondrial function [35], it will be intriguing to explore the differential mechanism of SIRT3 in response to MCL1 or BCL2/BCL2L1 suppression in future studies. Notably, ABZ is unable to upregulate the SIRT3 expression in K562/R cells. Given that ABT-263-resistant cells show MCL1 upregulation, it is apparent that the cellular context in K562 and K562/R cells differs. The differential expression of SIRT3 in ABZ-treated K562 and K562/R cells warrants further investigation.

Previous studies have revealed that MCL1 provides a survival benefit to MTA-treated cells and increases the lifespan of mitotically arrested cells [16,17]. Our data reveal that SIRT3 expression modulates the capability of ABZ to induce apoptosis in K562 cells when ABZ inhibits MCL1 expression. Nevertheless, it is worth noting that the death of K562 cells occurred after a prolonged treatment with ABZ (≥ 2 days, data not shown). Previous studies have shown that taxol-treated K562 cells show a delay in the mitotic apoptosis process [18]. Taken together, it can be deduced that SIRT3 upregulation delays, rather than abolishes, ABZ-induced apoptosis in K562 cells. Thus, the possibility that SIRT3 suppression immediately promotes ABZ-induced apoptosis could be considered. Previous studies have suggested that the blocking of SIRT3 activity can be utilized to improve the anti-leukemic efficacy of standard chemotherapeutic agents for acute myeloid leukemia [36]. Thus, further research to determine whether the combination of SIRT3 inhibitors improves the efficacy of MTAs on CML therapy may be useful.

## 4. Materials and Methods

### 4.1. Reagents

Without specific indication, the reagents obtained from Sigma-Aldrich Inc. (St. Louis, MO, USA) were used in this study, and cell culture supplements were the products of GIBCO/Life Technologies Inc. (Carlsbad, CA, USA). ABT-263 was obtained from Apexbio Technology LLC (Houston, TX, USA), and A-1210477 was the product of MedChem Express (Monmouth Junction, NJ, USA). MitoSOX Red, tetramethylrhodamine (TMRM), annexin V-FITC/propidium iodide (PI) apoptosis detection kit, and dichlorodihydrofluorescein diacetate (H_2_DCFDA) were obtained from Molecular Probes (Eugene, OR, USA); and Z-DEVD-FMK was from Calbiochem (San Diego, CA, USA).

### 4.2. Cell Culture

Human CML K562 and MEG-01 cells were obtained from BCRC (Hsinchu, Taiwan), and cultured in RPMI-1640 medium, supplemented with 10% FCS, 1% sodium pyruvate, 2 mM L-glutamine, penicillin (100 units/mL), and streptomycin (100 μg/mL).

For preparation of ABT-263-resistant K562 (K562/R) cells, parental K562 cells were exposed to 5 μM ABT-263 for 24 h. Following washing with PBS, the cells were cultured in FCS-containing medium for 2 days. After repeating the procedure 3 times, the resulting K562/R cells were maintained in FCS-containing medium, with the addition of 5 μM ABT-263. All cell lines were incubated in an incubator humidified with 5% CO_2_ atmosphere. Cell viability was evaluated using MTT assay. Apoptotic cell death was measured using annexin V-FITC/PI kit. The transfection of cells with pcDNA3-HA-Sp1 (described in [37]) or pCMV3-His-SIRT3 (Sino Biological Inc., Wayne, PA, USA) was performed using Lipofectamine according to manufacturer’s protocol (Thermo Fisher Scientific, Inc., Waltham, MA, USA).

### 4.3. Cell Cycle Analysis

Cell cycle analysis was conducted according to the method described in Wang et al. [15]. After staining with PI, cell cycle distribution of ABZ-treated cells was analyzed using flow cytometry.

### 4.4. Measurement of Intracellular ROS Levels and Mitochondrial Depolarization

After incubation of 10 μM H_2_DCFDA for 20 min, the intracellular ROS level of ABZ-treated cells was measured using a fluorescence microplate reader. MitoSOX Red probe was used to detect mitochondrial ROS level in ABZ-treated cells, and the MitoSOX Red-labeled cells were analyzed using flow cytometry. After incubation with 2 nM TMRM for 20 min, the mitochondrial membrane potential (ΔΨm) of ABZ-treated cells was measured using flow cytometric analysis. A reduction in TMRM fluorescence represented the loss of ΔΨm.

### 4.5. Preparation of Soluble and Insoluble Tubulin Fractions from Cells

Microtubule assembly assay was performed in essentially the same manner as described in Wang et al. [15]. The soluble (S) and insoluble tubulin (P) fractions were analyzed by immunoblotting with anti-α-tubulin antibody.

### 4.6. Quantitiative PCR (qPCR)

Total RNAs were isolated from cells using the RNeasy minikit (QIAGEN, Leiden, The Netherlands), and reverse transcribed into cDNAs using M-MLV reverse transcriptase (Promega, Madison, WI, USA). qPCR was conducted to detect the levels of MCL1, Sp1, and SIRT3 mRNA using GoTag qPCR Master mix (Promega, Madison, WI, USA). The primer sequences used are provided in Supplementary Table S1. To measure SIRT3 mRNA stability, the transcription of ABZ- or ABZ plus SB202190-treated cells was inhibited by incubation of actinomycin D (10 μg/mL) for 0.5, 1 and 2 h.

### 4.7. Immunoblot Analysis of Protein Expression

Primary antibody against caspase-3 was the product of Calbiochem (San Diego, CA, USA); and primary antibodies recognized MCL1, Sp1, and α-tubulin were obtained from Santa Cruz Biotechnology Inc. (Santa Cruz, CA, USA). Primary antibodies against ERK, p-ERK, PARP, CREB, p-CREB, STAT3, p-STAT3, SIRT3, NFκB/p65, p-NFκB/p65, p38 MAPK, p-p38 MAPK, Akt, p-Akt, GSK3β, p-GSK3β, BCL2, JNK, p-JNK, and β-actin were the products of Cell Signaling Technology (Beverly, MA, USA); antibodies against BCL2L1 were from BD Pharmingen Technical (San Jose, CA, USA). Total proteins were isolated from ABZ-treated cells using RIPA lysis buffer. Equal amount of proteins were separated on SDS-PAGE, followed by being electrophoretically transferred onto PVDF membranes. After blocking with 5% fat-free milk, the PVDF membranes were incubated with primary antibodies and then with the HRP-labeled secondary antibodies. Immunoreactive bands were visualized using a chemiluminescence solution (Perkin Elmer, Waltham, MA, USA). The immunoblots were repeated at least three times with similar results.

### 4.8. Luciferase Assay

The preparation of pGL3-MCL1 luciferase promoter construct was described in our previous studies [38]. A SIRT3 promoter containing nucleotides at positions −999 to 325 was subcloned into pGL3-basic vector (Promega), expressing the firefly luciferase. Transfection of the plasmids into leukemia cells was performed using Lipofectamine (Thermo Fisher Scientific, Inc.). Luciferase activity was quantified using the dual-luciferase reporter assay system (Promega, Madison, WI, USA).

### 4.9. Meaurement of SIRT3 Deacetylase Activity

The activity of SIRT3 deacetylase was measured using a SIRT3 Fluorimetric Drug Discovery kit (Enzo Life Sciences Inc., Farmingdale, NY, USA), as described previously [39]. Fluorescent intensity was measured using a fluorescence microplate reader, with excitation and emission wavelength at 360 nm and 460 nm, respectively. Activity was presented as a relative value compared with that of the control group.

### 4.10. Statistical Analysis

All data are presented as mean ± SD. Statistical analyses were conducted using two-tailed and Student’s *t*-test, and a *p* < 0.05 was considered statistically significant. All data presented are results obtained from at least three independent experiments.

## Supplementary Materials

The following are available online at https://www.mdpi.com/1422-0067/21/11/3907/s1, Supplementary Table S1. Primers used for qPCR.

## Abbreviations

ABZ	Albendazole
CML	Chronic myeloid leukemia
H_2_DCFDA	Dichlorodihydrofluorescein diacetate
FUZ	Flubendazole
MBZ	Mebendazole (MBZ)
ΔΨm	Mitochondrial membrane potential
MTA	Microtubule targeting agents
qPCR	Quantitiative PCR
TMRM	Tetramethylrhodamine
