# Supplementary material for: Albendazole-Induced SIRT3 Upregulation Protects Human Leukemia K562 Cells from the Cytotoxicity of MCL1 Suppression
Source: Int J Mol Sci. 2020 May 30;21(11):3907. doi: 10.3390/ijms21113907 (PMC7312678; doi:10.3390/ijms21113907)
Supplement: Supplementary file 1 [file ijms-21-03907-s001.pdf]

Supplementary Table S1. Primers used for qPCR.

Gene	Nucleotide sequence
MCL1 (forward)	5'-AAGAGGCTGGGATGGGTTTGTG-3'
MCL1 (reverse)	5'-TTGGTGGTGGTGGTGGTTGG-3'
Sp1 (forward)	5′- GAAAAAGGAGTTGGTGGCAATAAT-3′
Sp1 (reverse)	5′-AACTTGCTGGTTCTGTAAGTTGGG-3′
SIRT3 (forward)	5′-GACATTCGGGCTGACGTGATGGC-3′
SIRT3 (reverse)	5′-CAACCACATGCAGCAAGAACCTCTG-3′
GAPDH (forward)	5'-GAAATCCCATCACCATCTTCCAGG-3'
GAPDH (reverse)	5'-GAGCCCCAGCCTTCTCCATG-3'
